# Spatiotemporal Niche Differentiation of Ungulates in the Southwest Mountains, China

**DOI:** 10.3390/ani15233490

**Published:** 2025-12-03

**Authors:** Qingsong Jiang, Hangshu Xiao, Huaqiang Zhou, Ying Li, Jinghui Fu, Assan Meshach, Qiuxian Li, Liwen Kang, Li Yan, Yixin Shu, Jing Zhang, Zejun Zhang, Mingsheng Hong, Jianmei Xie

**Affiliations:** 1 Liziping Giant Panda’s Ecology and Conservation Observation and Research Station of Sichuan Province (Science and Technology Department of Sichuan Province), China West Normal University, Nanchong 637009, China; 18919552856@163.com (Q.J.); xhs2266@163.com (H.X.); 19161067250@163.com (H.Z.); 18086979870@163.com (Y.L.); jinghui7058@163.com (J.F.); meshachassan23@gmail.com (A.M.); 13388958049@163.com (Q.L.); liwenkang96@163.com (L.K.); 18884378798@163.com (L.Y.); shuyx1112@163.com (Y.S.); 18121850830@163.com (J.Z.); zhangzj@ioz.ac.cn (Z.Z.); 2 Key Laboratory of Southwest China Wildlife Resources Conservation (Ministry of Education), China West Normal University, Nanchong 637009, China; 3 College of Pharmacy, Chengdu University of Traditional Chinese Medicine, Chengdu 611137, China

**Keywords:** niche differentiation, ungulate, daily activity rhythm, spatial niche overlap index, sympatric coexistence

## Abstract

Spatiotemporal niche differentiation plays a critical role in facilitating mutual adaptation and sustaining coexistence among sympatric species. We investigated these patterns in sympatric ungulates through an infrared camera trap survey conducted in the Kazila Mountain region of southwestern China from July 2023 to May 2025. A total of seven species were recorded across 54 camera sites, with tufted deer (*Elaphodus cephalophus*) being the most frequently detected, while forest musk deer (*Moschus berezovskii*) and Chinese goral (*Naemorhedus griseus*) were the least. Nocturnality indices (β > 0.54 indicating nocturnal, β < 0.54 indicating diurnal, and β = 0.54 indicating no distinct diel preference) revealed significant differences in activity patterns among the five species. Tufted deer (β = 0.415), alpine musk deer (*Moschus chrysogaster*) (β = 0.438), and wild boar (*Sus scrofa*) (β = 0.234) were predominantly diurnal. In contrast, sambar (*Rusa unicolor*) (β = 0.571) was nocturnal, while the Chinese serow (*Capricornis milneedwardsii*) (β = 0.534) showed no strong diel preference. Nine of ten species pairs exhibited significant diel rhythm differences, with the exception of sambar-Chinese serow, and these rhythms showed marked seasonal variation, particularly in tufted deer, Chinese serow, and sambar. Temporal overlap was generally higher in the cold season for seven species pairs, suggesting that such overlap may be related to resource availability and increased interspecific competition under harsher conditions. Pianka’s overlap index (Oik) (ranging from 0 to 1, where 0 indicates no overlap and 1 indicates complete overlap) was used to assess spatial niche overlap, with values ranging from 0.16 (alpine musk deer–wild boar) to 0.86 (tufted deer–wild boar). Spatial autocorrelation and clustering analysis showed that tufted deer exhibited significant positive spatial autocorrelation, indicating a clustered high-value distribution, while the other species were randomly distributed. Spatial hotspot analysis revealed substantial overlap between tufted deer and wild boar, while the remaining species showed higher levels of spatial segregation. Collectively, these results suggest that seasonal variation in activity patterns, coupled with spatial segregation, mitigates interspecific competition and supports the stable sympatric coexistence of ungulates in this montane ecosystem.

## Simple Summary

Understanding how similar animal species share space and time is essential for maintaining biodiversity in mountain ecosystems. We used camera traps to study several species of wild ungulates living together in the Kazila Mountain area of southwestern China. From 2023 to 2025, seven species were recorded, including tufted deer, musk deer, goral, and wild boar. Each species showed distinct daily and seasonal activity patterns. For example, tufted deer and wild boar were mainly active during the day, while others showed no clear preference between day and night. These differences, together with spatial separation, help reduce competition for food and habitat. Our findings show that flexible activity rhythms and habitat use allow these species to coexist in the same environment, providing valuable insights for wildlife conservation and management in mountainous regions.

## 1. Introduction

The dynamics of competition and coexistence among sympatric species have long been central topics in community ecology and conservation biology, with profound implications for understanding interspecific interactions, population regulation, and ecological adaptation [1,2]. The competitive exclusion principle posits that species with highly similar ecological niches cannot stably coexist [3,4]. When species at the same trophic level exploit shared but limited resources, interspecific competition inevitably arises [5]. To alleviate excessive niche overlap and avoid competitive exclusion, sympatric species often partition resources across multiple ecological dimensions—including space, time, and diet—thereby promoting stable coexistence [6,7,8,9]. Such niche differentiation reduces interspecific competition, enhances resource-use efficiency, and contributes to the stability of community structure [8,10].

Among the various niche dimensions, the spatial and temporal components are considered the most fundamental [11]. Spatial and temporal patterns of habitat use and activity reflect the physiological constraints, ecological requirements, and interspecific interactions of species, and provide a critical framework for elucidating coexistence mechanisms and informing targeted biodiversity conservation. In this study, we define spatiotemporal niche differentiation as interspecific variation in habitat use and temporal activity patterns that reduces niche overlap and facilitates stable coexistence. Ungulates play key ecological roles in forest ecosystems, shaping vegetation structure and regeneration through foraging, trampling, and defecation, and influencing trophic dynamics and nutrient cycles [12,13,14,15,16,17]. In montane forest systems such as the Kazila Mountain region, where multiple ungulate species co-occur, understanding their ecological interactions and niche partitioning is essential for assessing community stability and guiding conservation.

Earlier studies on ungulates predominantly focused on single-species or single-dimension niche interactions [18,19,20,21,22,23,24]. However, in recent years, there has been a growing interest in multi-species and multi-dimensional approaches, addressing interactions across spatial, temporal, and dietary dimensions [25,26]. Despite this shift, relatively little attention has been given to interspecific interactions and seasonal variations in the spatiotemporal activity patterns of ungulates in high-altitude coniferous forests. Camera trapping technology has become a key tool for enhancing our understanding of wildlife interactions. It offers significant advantages, including extended monitoring durations, minimal disturbance to wildlife, and the ability to collect objective data on species distribution and activity patterns [27,28]. As a non-invasive method, camera trapping has been widely applied in ecological and conservation research, particularly in studies on species distribution, spatiotemporal niche differentiation, and biodiversity assessments. It is particularly well-suited for investigating interspecific interactions and seasonal variations in the activity patterns of ungulates in complex ecosystems.

Based on infrared camera-trap data collected from July 2023 to May 2025 in the Kazila Mountain region of southwestern China, this study investigated the composition and spatiotemporal distribution patterns of sympatric ungulates. Specifically, we aimed to: (1) identify daily activity rhythms and quantify the nocturnality of ungulates; (2) assess seasonal differences and overlaps in temporal activity patterns between cold and warm seasons; and (3) examine spatial niche differentiation and co-occurrence relationships among ungulate species. The findings provide new insights into spatiotemporal niche partitioning and coexistence mechanisms in this biodiverse montane ecosystem and offer valuable guidance for targeted conservation and sustainable management.

## 2. Materials and Methods

### 2.1. Study Area

**Figure 1:**
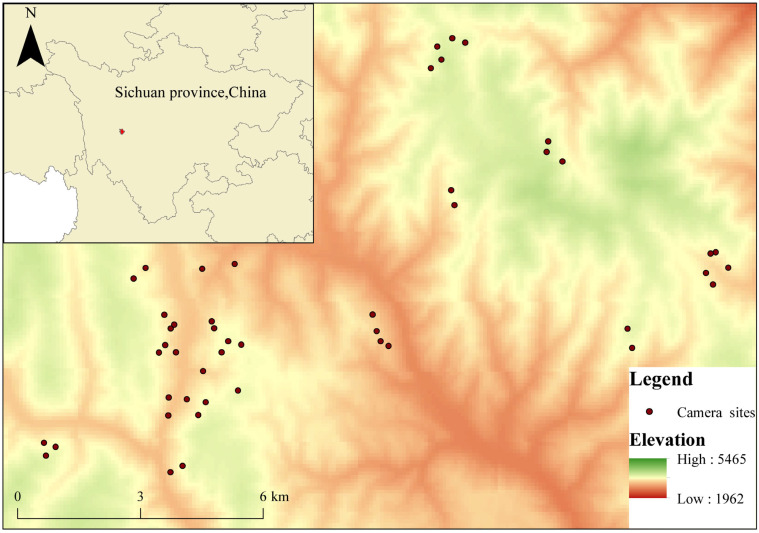
Infrared Camera Locations in the Kazila Mountain region of southwestern China.

The study area is located in Yajiang County, Ganzi Tibetan Autonomous Prefecture, Sichuan Province, situated on the southeastern edge of the Qinghai–Tibet Plateau, in the midsection of the Hengduan Mountains, between the Daxue and Shaluli mountain ranges (Figure 1). The region exhibits diverse vegetation types with distinct vertical zonation. The distribution is as follows: below 3000 m, deciduous broadleaf forests and mixed coniferous-broadleaf forests dominate; between 3000 and 4000 m, mixed coniferous-broadleaf forests, coniferous forests, and oak forests (e.g., Quercus species) are found; above 4000 m, alpine meadows predominate; above 4500 m, alpine screes and sparse vegetation on rocky flows are dominant. The area receives an average annual precipitation of 705 mm, with the rainy season concentrated between May and September. From November to April of the following year, the area is generally covered with snow. The Eruo River, a secondary tributary of the Yalong River, flows from north to south along the eastern side of Kazila Mountain. The region is rich in wildlife, home to several nationally protected ungulate species in China, including the forest musk deer, alpine musk deer, tufted deer, Chinese serow, Chinese goral, and sambar. For this study, we primarily conducted field research in the high-altitude core area of Kazila Mountain. The geographical coordinates of the study area range from 100°33′ E to 100°47′ E and 29°54′ N to 30°09′ N, with an elevation range of approximately 3100 to 4300 m, and an average altitude of around 3800 m.

### 2.2. Camera Trap Deployment

From July 2023 to May 2025, a total of 54 infrared camera (model LTI-6511, LTI Industrial Company Ltd., Shenzhen, China) traps were deployed in the Kazila Mountain region (see Figure 1). The locations of the camera traps were carefully selected based on a variety of natural factors and practical considerations to ensure the representativeness and scientific validity of the monitoring data. The selection of camera trap sites strictly followed ecological and geographical standards to accurately reflect the distribution of wildlife in the region.

The selection of camera trap locations took into account several factors, including habitat type, wildlife activity signs, geographical distribution, and topographical features. In terms of habitat type, areas with high canopy cover and open understory vegetation were specifically chosen, as these regions represent typical ecological environments of the area and facilitate coverage of different species’ activity spaces. Additionally, the cameras were placed in areas with frequent wildlife activity, such as animal corridors, water sources, or food resources, to ensure effective monitoring of various species. To avoid overlapping monitoring areas and ensure data independence, A distance of at least 500 m was maintained between most camera pairs, with the aim of minimizing the possibility of data duplication.

The cameras were installed on small- to medium-sized trees or shrubs at a height of 40–80 cm above the ground, and shrub and herbaceous vegetation within the monitoring area was cleared to reduce the risk of false triggers. For each camera station, geographic coordinates, habitat type, topographic features, distance to the nearest water source, and vegetation characteristics were recorded on-site using handheld GPS devices. The cameras were configured to capture three consecutive photos and record a 10 s video upon each trigger event, with a 10 s interval between triggers. Each image automatically recorded the date, time, and ambient temperature. Data were retrieved every 4 to 6 months, during which the batteries and memory cards were replaced, and cameras with poor image quality were adjusted as needed.

### 2.3. Data Analysis

#### 2.3.1. Nocturnality Index (β)

To minimize repeated detections, records of the same species captured at the same camera station within a 30 min interval were treated as a single independent detection event [29]. To assess animal nocturnal activity patterns, we defined the period from 18:00 to 06:00 as nighttime and from 06:00 to 18:00 as daytime. We then used the nocturnality index (β) to determine whether each ungulate species exhibited nocturnal or diurnal activity patterns. This method is based on the calculation principle of the relative abundance index [30], and the index was computed using the following formula:

$$\beta =\frac{D_{i}}{N_{i}}\times 100$$

where D_i_ is the number of independent valid photographs of species i taken during the nighttime period (18:00–06:00), and N_i_ is the total number of independent valid photographs of species i. β values > 0.54 indicate that the species is nocturnal. β values < 0.54 indicate diurnal activity. β values close to 0.54 suggest mixed activity with insignificant nocturnal behavior.

#### 2.3.2. Kernel Density Analysis

We applied the kernel density estimation (KDE) method to assess the overall daily activity rhythms of ungulate species throughout the year within the study area [31]. To eliminate differences in daily sunrise and sunset times across seasons, we converted detection times for each species from standard clock time to relative solar time [32]. We divided the year into a warm season (May–September) and a cold season (October to April of the following year), and analyzed daily activity rhythms separately for each season. We generated kernel density curves using the “overlap” (v 0.3.9) and “activity” (v 1.3.4) packages in R (v 4.2.3). In the resulting plots, the *x*-axis represents time, while the *y*-axis indicates activity intensity—the probability of detecting the target species at a given time point. We used the densityPlot() function to visualize daily activity rhythms for individual species, while the overlapPlot() function allowed us to compare daily activity patterns of ungulates between the warm and cold seasons.

We quantified the degree of overlap in daily activity rhythms between seasonal periods and among multiple species using the overlap index (Δ), calculated with the overlapEst() function. This index estimates the similarity in activity patterns between species, with values ranging from 0 to 1. Higher values indicate greater overlap, and a value of Δ = 1 denotes complete overlap between the activity patterns of two species. Following Ridout & Linkie’s method [31], we used Δ4 when both species had sample sizes ≥ 50, and Δ1 when the smaller sample size was <50. To improve reliability and assess differences in activity rhythms, we performed 1000 bootstrap resamplings using the compareCkern() function from the “activity” package [33]. We classified the degree of overlap as follows: Δ ≥ 0.8 indicates high overlap; 0.5 ≤ Δ < 0.8 indicates moderate overlap; and Δ < 0.5 indicates low overlap [34].

#### 2.3.3. Spatial Co-Occurrence Analysis

We used Pianka’s index (O_ik_) to evaluate spatial niche differentiation and coexistence among ungulate species. To account for variations in sampling effort across sites, we calculated the relative abundance index for each species at each site to assess spatial co-occurrence patterns. Pianka’s index (O_ik_) quantifies spatial niche overlap between species, ranging from 0 (no overlap) to 1 (complete overlap) [35]. We calculated the index using the following formula:

$$O_{\mathrm{ik}}=\frac{\sum_{j=1}^{r}(P_{\mathrm{ij}}P_{\mathrm{kj}})}{\sqrt{\sum_{j=1}^{r}P_{\mathrm{ij}}^{2}\sum_{j=1}^{r}P_{\mathrm{kj}}^{2}}}$$

The formula shows that P_ij_ and P_kj_ represent the ratio of independent detections of species i and species K at site j to the total independent detections of species. r refers to the total number of cameras.

Secondly, we used the number of independent valid detections as an indicator of activity intensity. To account for the uneven spatial distribution of camera traps, we first performed a kernel density analysis on all camera locations to obtain a local trap-density value for each station. We then standardized the activity intensity of each species at each station by dividing the number of independent detections by the corresponding trap-density value, yielding a trap-density-corrected activity intensity. We subsequently used this corrected activity intensity for spatial analyses.

To visualize the spatial distribution patterns, we used ESRI ArcGIS 10.8 to interpolate the corrected activity intensity values using the Inverse Distance Weighting (IDW) method. We conducted spatial statistical analyses using the Spatial Statistics Tools in ArcGIS, including global spatial autocorrelation analysis (Global Moran’s I) to examine overall spatial dependence [36], and high/low clustering analysis (Getis-Ord General G) to identify global clustering trends. Finally, we performed hotspot analysis (Getis-Ord Gi) using the Mapping Clusters tool to detect local clusters of high and low activity intensity for each species across the study area [37].

## 3. Results

### 3.1. Camera Monitoring Results

A total of 27,570 camera-days were accumulated from July 2023 to May 2025, yielding 6818 independent wildlife detections, of which 3291 belonged to ungulates (Table 1). Seven ungulate species were recorded, including two Class I National Protected Species in China (forest musk deer and alpine musk deer) and four Class II National Protected species in China (tufted deer, Chinese serow, sambar, and Chinese goral). Among all species, tufted deer showed a much higher detection frequency than the others, whereas Chinese goral and forest musk deer were rarely recorded. Due to their extremely small sample sizes, these two species were excluded from subsequent analyses.

### 3.2. Temporal Niche Differentiation Among Ungulate Species

**Figure 2:**
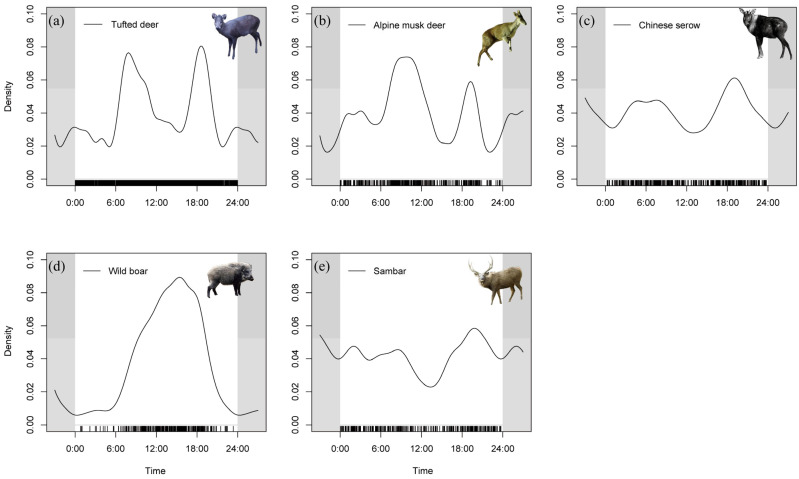
Daily Activity Rhythm of Ungulates. (**a**) Tufted deer, (**b**) Alpine musk Deer, (**c**) Chinese serow, (**d**) Wild boar, (**e**) Sambar.

Based on the nocturnality analysis, tufted deer (β = 0.415), alpine musk deer (β = 0.438), and wild boar (β = 0.234) were identified as typical diurnal species. Both tufted deer and alpine musk deer exhibited distinct bimodal activity patterns. Tufted deer showed activity peaks at approximately 08:00 and 19:00 (Figure 2a), while alpine musk deer peaked around 09:00 and 19:00 (Figure 2b). In contrast, wild boar displayed a unimodal daily activity pattern, with a peak around 15:00 (Figure 2d). The Chinese serow (β = 0.534) showed no clear difference between daytime and nighttime activity, with activity distributed evenly throughout the 24 h cycle. It exhibited two activity peaks, one between 04:00 and 08:00 and another around 19:00 (Figure 2c). The sambar (β = 0.571) is nocturnal, with three minor activity peaks at approximately 02:00, 09:00, and 20:00, along with a noticeable trough around 12:00 (Figure 2e).

**Figure 3:**
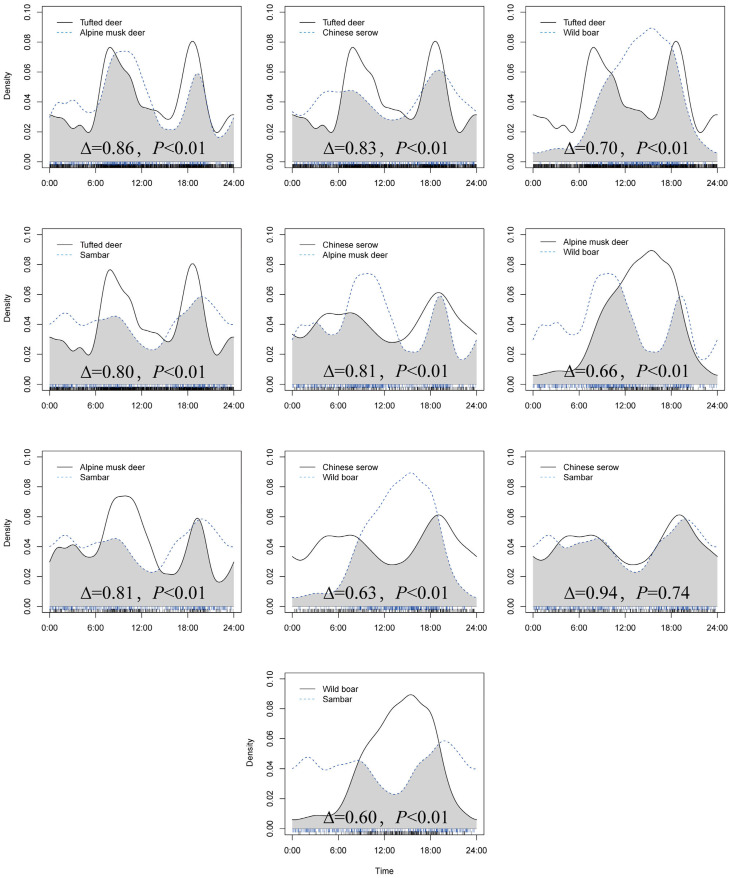
Comparison of Daily Activity Rhythm Differences Among Ungulate Species.

All species pairs exhibited varying degrees of overlap in their activity rhythms (Figure 3). The highest degree of overlap was observed between Chinese serow and sambar (∆ = 0.94, *p* > 0.05, Figure 3), indicating highly similar daily activity patterns. In contrast, the lowest overlap occurred between sambar and wild boar (∆ = 0.60, *p* < 0.01, Figure 3), whose activity rhythms differed substantially—wild boar’s activity peak coincided with the activity trough of sambar. For all other species pairs, daily activity rhythms differed significantly (*p* < 0.01).

### 3.3. Seasonal Differentiation Among Ungulate Species

**Figure 4:**
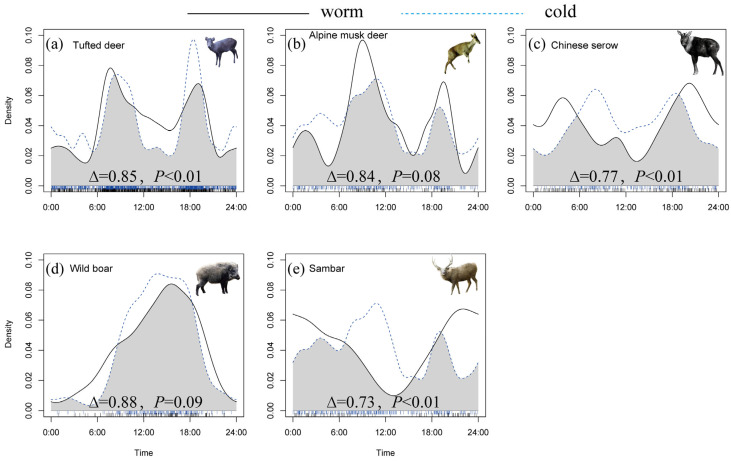
Comparison of Daily Activity Rhythm Differences of Ungulate Species Between Cold and Warm Seasons. (**a**) Tufted deer. (**b**) Alpine musk deer. (**c**) Chinese serow. (**d**) Wild boar. (**e**) Sambar.

Seasonal comparisons revealed clear differences in daily activity rhythms among the ungulate species (Figure 4). Tufted deer, Chinese serow, and sambar exhibited significant seasonal shifts, whereas alpine musk deer and wild boar showed broadly consistent activity patterns across seasons.

Across species, seasonal changes were mainly reflected in the relative timing and intensity of morning and evening activity peaks. Tufted deer maintained a bimodal pattern year-round, but both peaks shifted toward later morning and earlier evening during the cold season, accompanied by reduced midday activity. Alpine musk deer also retained a consistent tri-modal pattern, with all peaks occurring slightly later in the cold season. Chinese serow displayed the most pronounced temporal shift, with both morning and evening peaks delayed during the cold season and daytime activity becoming more prominent. Wild boar preserved a unimodal pattern across seasons, though the timing of the single peak advanced in the cold season. Sambar showed the strongest seasonal contrast, shifting from a mainly nocturnal pattern in the warm season to a broadly diurnal distribution with two daytime peaks in the cold season.

We found notable differences in overlap patterns between the cold and warm seasons within the study area (Table 2). Overall, inter-species daily activity overlap was generally higher in the cold season. Among the ten species pairs formed by the five ungulate species, all pairs exhibited moderate to high overlap in both seasons. However, during the warm season, only two species pairs showed high overlap, while in the cold season, six species pairs exhibited high overlap. Additionally, in seven of the ten species pairs, the overlap index was higher in the cold season than in the warm season. Only three species pairs had higher overlap values in the warm season compared to the cold season.

### 3.4. Spatial Niche Differentiation Among Ungulate Species

**Figure 5:**
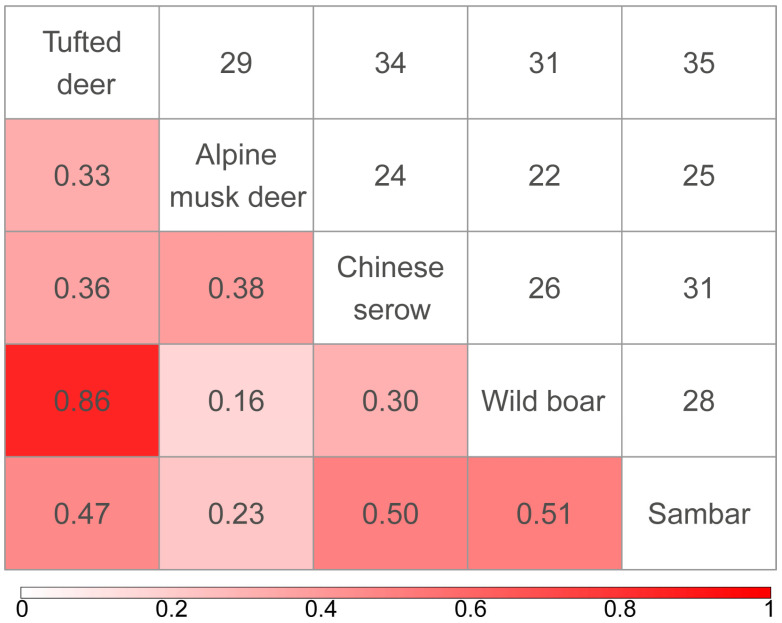
Spatial Niche Overlap among Ungulate Species. The lower-left diagonal shows the species’ niche overlap index (Pianka’s Index), and the upper-right diagonal shows the number of co-occurring sites of the species.

The spatial co-occurrence analysis of ungulate species in the study area revealed that spatial niche overlap indices (O_ik_) among the five species ranged from 0.16 to 0.86 (Figure 5). The highest spatial niche overlap occurred between tufted deer and wild boar (*n* = 31, O_ik_ = 0.86), while the lowest was observed between alpine musk deer and wild boar (*n* = 22, O_ik_ = 0.16).

No significant spatial autocorrelation was detected in the activity intensity of the four ungulate species, other than tufted deer, with their distributions randomly dispersed across the study area (Table 3). In contrast, tufted deer exhibited significant positive spatial autocorrelation, with Moran’s I = 0.3055, Z = 2.4347, and *p* = 0.0149, indicating a spatially clustered distribution pattern. Further analysis using the Getis-Ord General G statistic (General G = 0.0296, Z = 2.3760, *p* = 0.0175) confirmed that the activity intensity of tufted deer formed high-value spatial clusters, suggesting a pronounced aggregation in their spatial distribution (Table 3).

**Figure 6:**
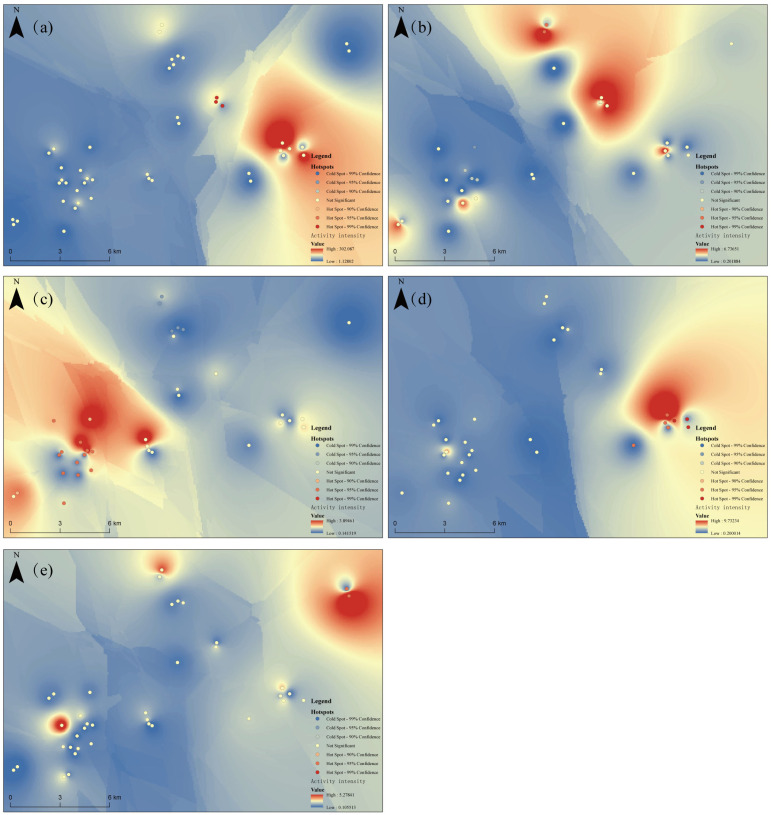
Activity Hotspot Analysis of Ungulate Species. (**a**) Tufted deer. (**b**) Alpine musk deer. (**c**) Chinese serow. (**d**) Wild boar. (**e**) Sambar.

Further spatial hotspot analysis showed that all ungulate species exhibited some degree of spatial differentiation in their activity hotspots (Figure 6). Although the hotspot locations varied among species, tufted deer and wild boar displayed a high degree of similarity in their overall activity-intensity distribution patterns, indicating substantial spatial co-occurrence across general use areas. In contrast, the remaining species pairs showed largely segregated spatial distribution patterns, suggesting lower levels of spatial co-occurrence within their core high-use areas.

## 4. Discussion

### 4.1. Daily Activity Rhythms

The temporal separation of activity across diurnal, crepuscular, and nocturnal periods is recognized as an important mechanism for reducing competition and promoting species coexistence [38]. Diel activity rhythms are regulated by the combined influence of endogenous biological clocks and external factors such as genetic traits and seasonal variation [39]. Ungulate species commonly display substantial variation in diel activity patterns across different spatial scales [40].

In this study, pronounced interspecific differences in diel activity rhythms were observed among the focal ungulate species. Except for Chinese serow and sambar, most species pairs exhibited significant differences in their activity patterns, suggesting that closely related species may undergo temporal niche differentiation when sympatrically distributed, driven by differences in environmental adaptation and interspecific competition [41].

Comparisons between our findings and studies from other regions revealed both similarities and discrepancies. The crepuscular bimodal activity patterns of tufted deer and alpine musk deer were consistent with observations from the Gongga Mountain region in Sichuan [24], indicating that crepuscular activity may represent a common behavioral strategy among forest-dwelling ungulates in the mountainous regions of southwestern China. The dawn-and-dusk peaks of Chinese serow were also consistent with findings from Fanjingshan [42], and the unimodal diurnal activity of wild boar aligned with reports from Fanjingshan and other mountainous areas of southwestern China [42,43]. However, several species in this study exhibited diel activity patterns distinct from those reported elsewhere. For instance, tufted deer showed a unimodal diurnal pattern in Fanjingshan [42], whereas alpine musk deer in Tibet were predominantly nocturnal [44]. In this study, Chinese serow exhibited no pronounced activity peaks and maintained activity throughout the day, unlike the afternoon and midnight peaks reported from Gaoligong Mountain [45] or the predominantly diurnal activity observed in the Qinling Mountains [26]. Sambar displayed a three-peak diel pattern, which differed from the bimodal pattern documented in Gongga Mountain and the unimodal nocturnal pattern observed in southwestern China [24,43]. In addition, studies from Europe and the Mediterranean have commonly characterized wild boar as nocturnal [46,47,48], contrasting sharply with the diurnal tendency observed in our study area. These regional discrepancies underscore the strong plasticity of ungulate diel activity rhythms.

Multiple ecological factors may account for such variation. First, environmental heterogeneity—including differences in climate, elevation, vegetation structure, and seasonal resource availability—can strongly influence the activity budgets of ungulates [49,50]. Second, regional differences in community composition and interaction networks—such as variation in predator distributions, interspecific competition, and levels of human disturbance—can reshape diel activity strategies [51,52]. Moreover, human disturbance itself is an important driver of wildlife activity rhythms [53]. The Kazila Mountain region is characterized by steep terrain, dense forest cover, and pronounced seasonality, and these unique ecological conditions likely shape the temporal strategies of local ungulate populations.

### 4.2. Seasonal Differences in Daily Activity Rhythms

Changes in resource availability and climatic conditions can drive seasonal shifts in activity patterns, reflecting the intensity of interspecific competition among sympatric species [54,55]. Our analysis of seasonal daily activity patterns showed that tufted deer, Chinese serow, and sambar exhibited significant differences between the cold and warm seasons, while alpine musk deer and wild boar showed no significant seasonal variation. For all species, activity peaks shifted across seasons: morning peaks were delayed, and evening peaks occurred earlier in the cold season. These shifts suggest that ungulates adjust their activity schedules to cope with seasonal environmental changes.

Two primary mechanisms may explain these seasonal shifts: (1) Seasonal factors, such as temperature, precipitation, and food availability, have cumulative effects on animal physiology, movement ecology, foraging strategies, and survival [26,56,57]. In the cold season, lower nighttime and crepuscular temperatures, combined with reduced food quality, may constrain activity. In contrast, higher midday temperatures prompt animals to concentrate their activity during warmer periods, minimizing the energetic costs of thermoregulation. (2) In the cold season, limited availability of local edible fungi, such as cordyceps and matsutake, and reduced human disturbance due to winter herding practices may create more favorable conditions for ungulates to be active around midday.

The seasonal analysis of temporal niche overlap revealed higher daily activity overlap in the cold season compared to the warm season. For species pairs excluding Chinese serow and sambar, the overlap indices generally decreased in the cold season, suggesting niche separation in response to intensified competition. However, pairs involving Chinese serow and sambar showed higher overlap in the cold season, possibly due to reduced nocturnal foraging opportunities, leading to a shift in daily activity towards the daytime to adapt to temperature fluctuations [58,59]. This shift could increase competition with primarily diurnal species. Additionally, the consistently high activity overlap between tufted deer and alpine musk deer, and between sambar and Chinese serow, suggests that temporal niche partitioning is not the main mechanism driving coexistence for these species pairs. Instead, spatial or trophic niche differentiation may help mitigate competition and support their coexistence.

### 4.3. Spatial Co-Occurrence Patterns

Different species vary in their resource selection and habitat use due to the combined influence of multiple ecological factors; therefore, daily activity rhythms alone cannot fully explain interspecific interactions [60,61]. Spatial partitioning is likewise an important mechanism that mitigates competition and facilitates long-term coexistence among sympatric species [62].

Interspecific differences in habitat selection can effectively reduce competition for space and other environmental resources, thus promoting the coexistence of sympatric species [63]. In this study, except for tufted deer and wild boar, the other species exhibited relatively low spatial overlap. Such significant differences in habitat selection help minimize their competition for the same resources, effectively reducing interspecific competition and facilitating stable coexistence. In contrast, tufted deer and wild boar showed higher spatial niche overlap, indicating potential habitat competition between the two species. However, their long-term coexistence in the region suggests that differences in other ecological dimensions may mitigate the competitive pressure associated with spatial overlap. First, their temporal overlap was moderate; therefore, even when using the same areas, they did not always utilize them at the same time, and partial temporal segregation may reduce direct encounters and immediate competition. Secondly, wild boar exhibit broad omnivory and high ecological plasticity [26,64], allowing them to exploit a wide range of resources not utilized by tufted deer, further reducing the potential for resource competition.

Taken together, even when species exhibit substantial spatial overlap, differences in temporal activity and resource-use strategies can still reduce direct competition and promote coexistence. This highlights that spatial niche overlap should not be interpreted in isolation; rather, it must be considered alongside differentiation in other niche dimensions to more fully understand the coexistence mechanisms of sympatric species.

## 5. Conclusions

Spatiotemporal niche differentiation plays a crucial role in shaping behavioral adaptation and facilitating long-term coexistence among sympatric ungulates. Based on infrared camera-trap data, this study systematically examined the temporal and spatial niche relationships of five ungulate species in the Kazila Mountain region. Overall, the species exhibited varying degrees of differentiation in daily activity rhythms, seasonal behavioral adjustments, and spatial habitat use, and such multidimensional niche partitioning effectively reduced potential competitive pressure. Even where spatial overlap occurred, differences in temporal activity and resource-use strategies buffered direct competition, supporting the stable coexistence of these sympatric ungulates. These findings provide essential baseline information on interspecific interactions and offer a scientific reference for habitat management and conservation planning in the region.

## Figures and Tables

**Table 1:** Composition of Ungulate Species and Independent Detection Count.

Species	The Number of Independent Detections		
	Cold Season	Warm Season	Total
Tufted deer (*Elaphodus cephalophus*)	888	1072	1960
Alpine musk deer (*Moschus chrysogaster*)	235	114	349
Chinese serow (*Capricornis milneedwardsii*)	169	179	348
Wild boar (*Sus scrofa*)	174	129	303
*Sambar (Rusa unicolor*)	199	96	296
Forest musk deer (*Moschus berezovskii*)	23	6	29
Chinese goral (*Naemorhedus griseus*)	1	5	6

**Table 2:** Overlap of daily Activity Rhythms Among Five Ungulate Species in Kazila Mountain During the Cold and Warm Seasons.

Period	Species	Tufted Deer	Alpine Musk Deer	Chinese Serow	Wild Boar	Sambar
Cold season	Tufted deer	—	*p* < 0.01	*p* = 0.027	*p* < 0.01	*p* < 0.01
	Alpine musk deer	Δ = 0.83	—	*p* < 0.01	*p* < 0.01	*p* = 0.018
	Chinese serow	Δ = 0.85	Δ = 0.83	—	*p* < 0.01	*p* = 0.447
	Wild boar	Δ = 0.62	Δ = 0.63	Δ = 0.66	—	*p* < 0.01
	Sambar	Δ = 0.83	Δ = 0.84	Δ = 0.91	Δ = 0.64	—
Warm season	Tufted deer	—	*p* = 0.199	*p* < 0.01	*p* < 0.01	*p* < 0.01
	Alpine musk deer	Δ = 0.87	—	*p* < 0.01	*p* < 0.01	*p* < 0.01
	Chinese serow	Δ = 0.72	Δ = 0.69	—	*p* < 0.01	*p* = 0.491
	Wild boar	Δ = 0.78	Δ = 0.70	Δ = 0.61	—	*p* < 0.01
	Sambar	Δ = 0.65	Δ = 0.64	Δ = 0.88	Δ = 0.52	—

**Table 3:** Spatial Autocorrelation and Cluster Analysis of Activity Intensity for Each Species.

Species	Spatial Autocorrelation Analysis			Cluster Analysis		
	Moran’s I	Z-Value	*p*-Value	General G	Z-Value	*p*-Value
Tufted deer	0.3055	2.4347	0.0149	0.0296	2.3760	0.0175
Chinese serow	0.1124	1.5684	0.1168	0.0003	1.3452	0.1786
Wild boar	0.0784	1.5256	0.1271	0.0492	1.7648	0.0622
Sambar	−0.1215	−0.7603	0.4471	0.0235	−0.7767	0.4374
Alpine musk deer	−0.0275	0.0789	0.9373	0.0382	0.4370	0.6621

## Data Availability

The camera-trapping data are owned by the China West Normal University and are authorized for use in this study.
